# Regulation of Bestrophins by Ca^2+^: A Theoretical and Experimental Study

**DOI:** 10.1371/journal.pone.0004672

**Published:** 2009-03-05

**Authors:** Agata Kranjc, Federico W. Grillo, Juraj Rievaj, Anna Boccaccio, Fabio Pietrucci, Anna Menini, Paolo Carloni, Claudio Anselmi

**Affiliations:** 1 International School for Advanced Studies (SISSA/ISAS), Trieste, Italy; 2 Italian Institute of Technology (IIT), SISSA-Unit, Trieste, Italy; 3 CNR-INFM-DEMOCRITOS Modeling Center for Research in Atomistic Simulation, Trieste, Italy; Griffith University, Australia

## Abstract

Bestrophins are a recently discovered family of Cl^−^ channels, for which no structural information is available. Some family members are activated by increased intracellular Ca^2+^ concentration. Bestrophins feature a well conserved Asp-rich tract in their COOH terminus (Asp-rich domain), which is homologous to Ca^2+^-binding motifs in human thrombospondins and in human big-conductance Ca^2+^- and voltage-gated K^+^ channels (BK_Ca_). Consequently, the Asp-rich domain is also a candidate for Ca^2+^ binding in bestrophins. Based on these considerations, we constructed homology models of human bestrophin-1 (Best1) Asp-rich domain using human thrombospondin-1 X-ray structure as a template. Molecular dynamics simulations were used to identify Asp and Glu residues binding Ca^2+^ and to predict the effects of their mutations to alanine. We then proceeded to test selected mutations in the Asp-rich domain of the highly homologous mouse bestrophin-2. The mutants expressed in HEK-293 cells were investigated by electrophysiological experiments using the whole-cell voltage-clamp technique. Based on our molecular modeling results, we predicted that Asp-rich domain has two defined binding sites and that D301A and D304A mutations may impact the binding of the metal ions. The experiments confirmed that these mutations do actually affect the function of the protein causing a large decrease in the Ca^2+^-activated Cl^−^ current, fully consistent with our predictions. In addition, other studied mutations (E306A, D312A) did not decrease Ca^2+^-activated Cl^−^ current in agreement with modeling results.

## Introduction

In 1998, the gene VMD2, which encodes for the human bestrophin-1 (hBest1) protein, was found to be responsible for the inherited Best vitelliform macular dystrophy. This is an early-onset autosomal dominant maculopathy typically characterized by yellowish lesions in the central area of the retina [1], [2]. Since then, four human (hBest1-4) and three mouse (mBest1-3) genes were identified, together with other genes from different species (for review, see [3]).

All human and mouse members of the bestrophin family have been expressed in heterologous systems [4]–[8]. The proteins form Cl^−^ channels, likely to be composed by several subunits [4], [9]. Three of them (hBest1, hBest4 and mBest2) are regulated by Ca^2+^ [4], [5], [10], [11]. They have been proposed as the putative molecular counterpart of the Ca^2+^-activated Cl^−^ current in some epithelial cells [12]–[14] and in olfactory sensory neurons (mBest2), where they were suggested to play a pivotal role for the olfactory transduction [11], [15], [16]. However, recent studies indicated that the TMEM16 family represents a new candidate molecular counterpart of Ca^2+^-activated Cl^−^ channels [17].

Although the link between the protein and the Best disease has not been clarified yet [3], [18], it has been established that more than one hundred mutations, mostly located in hydrophobic domains, are associated with the disease [19], [20]. Some functionally highly relevant mutations (Q293K/H, L294V, ΔI295, G299E/R, E300D, D301E, T307I and D312N) [3], [4], [21]–[23] are located at the C-terminal domain inside the Asp-rich sequence from W287 to D323 (Asp-rich domain, hereafter). These mutations reduce whole-cell currents compared to wild-type (WT) hBest1 after their expression in heterologous systems [3], [4], [21]–[23]. Furthermore, inserting a stop codon in position L294 (but not in position F353) causes a strong decrease of the current [24].

Clearly, knowledge of the structural determinants of the protein is crucial to understand its function both in health and in disease conditions. However, no 3-D structural information is available so far. Nevertheless, two topology models have been advanced [7], [25]. According to them, the N- and C-terminal domains of bestrophins would be located at the intracellular side of the membrane and would be connected to four [25] or five [7] hydrophobic domains forming the channel.

In an effort to characterize prominent structural and functional features of bestrophins, we noticed that the Asp-rich domain, which has been indicated as a possible Ca^2+^ sensor for bestrophins [10], is the most amenable region for molecular modeling studies. In fact, it has significant sequence similarity with other domains for which structural information is available. These include Ca^2+^-binding domains as the cytoplasmic Ca^2+^ bowl motif [26], [27] of the big conductance Ca^2+^- and voltage-gated K^+^ channels (BK_Ca_) [26]–[30] and the type 3 (T3) motifs in the C-terminal region of thrombospondins [31]. The first corresponds to a highly conserved 28 amino acid long peptide [26], [27] characterized by a net negative charge and five adjacent Asp's residues (D959–D963 in human BK_Ca_). Site-directed mutagenesis experiments have confirmed the role of Ca^2+^ bowl in the binding of calcium ions [30], [32]. Although the experimental 3-D structure is not available, a theoretical model of this domain in BK_Ca_ channels has been recently provided by us [33]. The model turned out to be consistent with most of the available experimental data [30], [32].

The second type of Ca^2+^-binding domains, T3 repeats of thrombospondins, consists in tandems of Asp-rich motifs, which resemble EF hands in the spacing of acidic side chains [34], [35]. The X-ray structures of the C-terminal fragments of two human thrombospondins (TSP-1 and TSP-2) show that they comprise several T3 motifs bound to as much as twenty six Ca^2+^ ions [36], [37].

Because of the significant sequence similarity between the Asp-rich domain and the aforementioned Ca^2+^-binding domains, it is plausible to hypothesize that Ca^2+^ activation of bestrophins could involve, at least in part, Ca^2+^-binding to the Asp-rich domain. This hypothesis is fully consistent with the experimental evidence from mutations of amino acids constituting putative Ca^2+^ ligands (E300D, D301E and D312N) in the domain, which affect dramatically the measured current in the HEK-293 cells [4], [22]. In addition, inserting a stop codon at position F353, located after the Asp-rich domain, does not cause a current reduction, while cutting the channel at the beginning of the Asp-rich domain (L294) significantly reduce the current [24]. Therefore, we focused our attention on the Asp-rich domain, whereas other possible Ca^2+^-binding domains were not explored here. On the other hand, computer simulations of the entire channel, which would help to clarify how Ca^2+^ binding could affect channel gating, are not feasible due to the lack of structural information of the transmembrane region of the channel.

A straightforward approach to test if Asp-rich domain is involved in Ca^2+^ activation of bestrophins would be to mutate randomly its Asp/Glu residues and measure the functional properties of the channel. Here instead we followed a more rational approach, which uses computation to guide the experiments. We first constructed a structural model of hBest1 Asp-rich domain based on the TSP-1 T3_6_ X-ray structure [36], which has the largest number of Ca^2+^-binding residues conserved across bestrophins. The models of WT and selected alanine mutants were then studied by molecular simulations. Mutations with the largest predicted effect on Ca^2+^ binding turned out to cause a dramatic decrease in the experimentally measured mBest2 Ca^2+^-activated current (more than 96% reduction with respect to the WT mBest2). The consistency between theory and experiments permits to have the first structural insights on the hBest1 protein.

## Results

Our strategy to identify Asp and Glu amino acids important for the function of hBest1 was twofold. First, we attempted to identify residues that may bind the Ca^2+^ ions in the WT. Next, we predicted the effects of mutations to alanine of these residues on the structure and function of the Asp-rich domain. The effect is non-trivial, because residues, which bind Ca^2+^ ions in the WT, can be replaced by others when mutated to alanine.

### Identification of acidic residues binding Ca^2+^ in hBest1 Asp-rich domain

**Figure 1:**
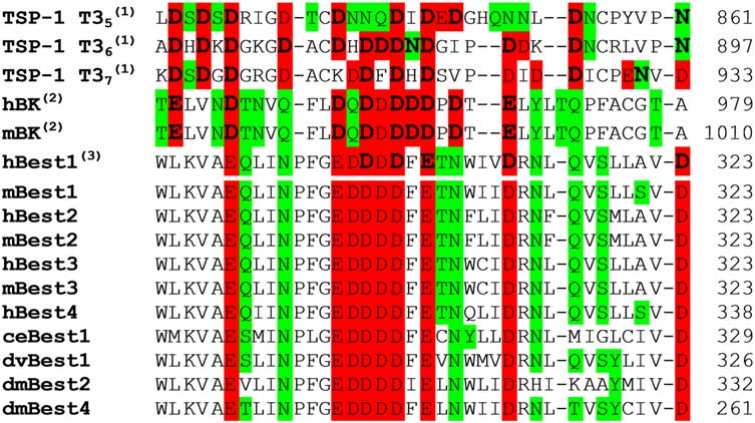
Alignment of the Asp-rich sequences from TSP-1, BK_Ca_ and bestrophins. Alignment between TSP-1 T3_5–7_ repeats [36], human BK_Ca_ Ca^2+^ bowl (SwissProt entry: Q12791) [29], mouse BK_Ca_ Ca^2+^ bowl (SwissProt entry: Q08460) [28] and the Asp-rich domain at the C-terminal part of hBest1 [1], [2] (SwissProt entry: O76090). hBest1 shares in this tract 33 to 36% of sequence similarity with TSP-1 T3_5–7_ [36] motifs and 42% with BK_Ca_ Ca^2+^ bowl [30]. Bold letters represent residues, which are involved in Ca^2+^ binding in TSP-1 (as found in the crystal structure^(1)^ [36]), hBK_Ca_ and mBK_Ca_ (from the model in a previous work^(2)^ [33]) and hBest1 (this work^(3)^). O-donor residues are colored in red (negatively charged residues) or green (neutral residues). The alignments of selected tracts from the C-terminal domains of other bestrophins are also shown: mBest1 (mouse bestrophin-1, SwissProt entry: O88870), hBest2 (human bestrophin-2, SwissProt entry: Q8NFU1), mBest2 (mouse bestrophin-2, SwissProt entry: Q8BGM5), hBest3 (human bestrophin-3, SwissProt entry: Q8N1M1), mBest3 (mouse bestrophin-3, SwissProt entry: Q6H1V1), hBest4 (human bestrophin-4, SwissProt entry: Q8NFU0), ceBest1 (*Caenorhabditis elegans* bestrophin-1, SwissProt entry: Q21973), dvBest1 (*Drosophila virilis* bestrophin-1, SwissProt entry: Q20C62), dmBest2 (*Drosophila melanogaster* bestrophin-2, SwissProt entry: Q9VRW4), dmBest4 (*Drosophila melanogaster* bestrophin-4, SwissProt entry: Q9VUM6).

**Figure 2:**
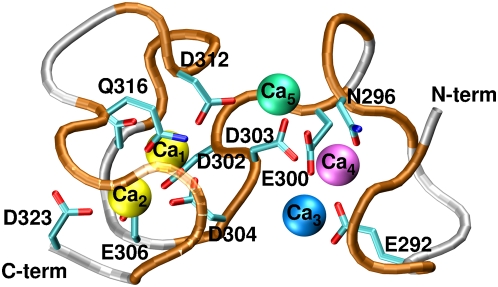
Initial model of the Asp-rich domain studied by the MD simulations. M5 comprises all five Ca^2+^ ions (shown as spheres). M4 model is the same as M5 but without Ca_3_ (blue). M3′ is the same as M5, but without Ca_3_ (blue) and Ca_5_ (green), while in M3″ Ca_3_ (blue) and Ca_4_ (magenta) were excluded. Asp/Glu/Asn and Gln residues coordinating Ca^2+^ ions are shown. Ca^2+^ ions are labeled for clarity as Ca_1–5_ according to their binding positions. The same labels are used throughout the text.

The structural determinants of the Asp-rich domain were modeled by using the X-ray structure of TSP-1 T3_6_ repeat [36] as a template, because it exhibits the largest number of Ca^2+^-binding residues conserved across bestrophins family (Figure 1). The number of Ca^2+^ ions bound to the Asp-rich domain is unknown. Typically, as observed from other Ca^2+^-binding domains in the PDB database, each Ca^2+^ ion is usually coordinated by three Asp/Glu residues [38]. Since the template has five Ca^2+^ ions bound and 33% of negatively charged residues more than the Asp-rich domain, it is plausible to expect that the last would bind five or less Ca^2+^ ions. Therefore we considered models including five (M5), four (M4), and three (M3′, M3″) Ca^2+^ ions (Figure 2).

**Figure 3:**
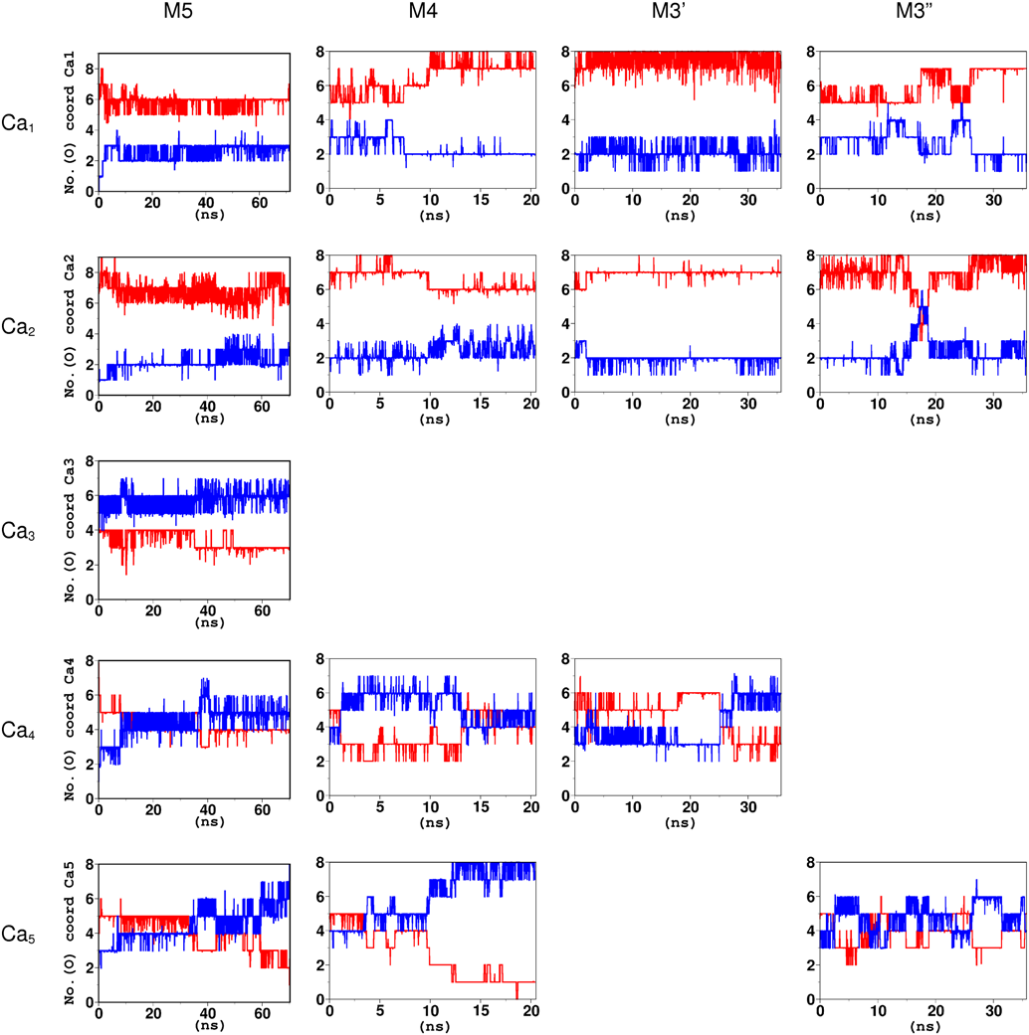
MD simulations of bestrophin Asp-rich domain models. Ca^2+^ coordination numbers contributed by protein O-donors (red) and water molecules (blue) plotted as a function of simulation time. Ca^2+^ ions were defined as stably bound if their coordination number was at least 6 and not more than 2 water molecules contributed to their binding.

In all molecular dynamics (MD) simulations, two ions (Ca_1_ and Ca_2_ in Figure 3) remained bound. Ca_1_ was bound to D302, D304, E306 and D312, Ca_2_ to D303, D304, E306, D312, Q316, S318 and D323 (Table S1). Instead, the other ions were partially or completely solvated. The decrease in the number of bound Ca^2+^ ions with respect to TSP-1 T3_6_ can be caused by a combination of factors: first of all, the lower number of negatively charged residues in Asp-rich domain with respect to T3_6_ (Figure 1), but also the limitations associated with the force field (see Text S1).

MD simulations showed that residues D302, D304, E306, D312 and D323 (Table S1) play a major role for Ca^2+^ binding. In order to identify other Ca^2+^-binding residues, we used an enhanced sampling method, *i.e.* metadynamics [39]. In this approach, the MD simulation is altered by biasing the selected collective variables (CVs) with a history-dependent potential. The bias, constructed as a sum of Gaussians centered along the trajectory of the system, reduces the free-energy barriers and enhances the rate of transitions, in the present case the binding and unbinding of Ca^2+^ ions.

The starting structure for the metadynamics simulation was selected among our energy-minimized models. We chose M3′ model, because it kept the largest number of bound Ca^2+^ ions (three) during the MD simulations for as long as 25 ns (Figure 3). The selected CVs measured the extent of binding of Ca_2_ and Ca_4_, respectively, since Ca_1_ was always bound to a well-defined binding pocket in all previous simulations. The limitations of the force field along with the large number of conformational degrees of freedom of the system prevented us from calculating the free-energy profile associated to Ca^2+^ binding. Therefore, the simulation was only used to sample stable configurations, different from those studied in the MD.

**Figure 4:**
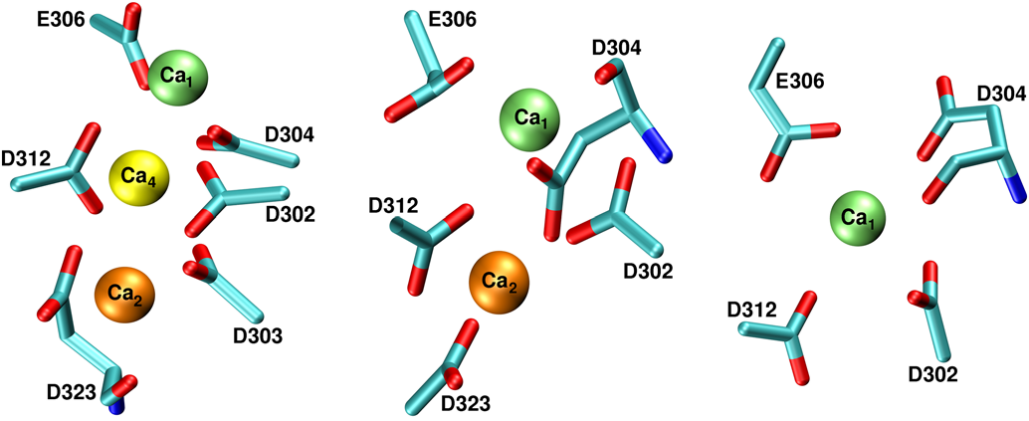
Metadynamics simulations of M3′ model. Pictorial representation of the most populated structures with one, two or three coordinated Ca^2+^ ions. Ca_1_ binding pocket formed by D302, D304, E306 and D312 is always occupied. In addition, when a second Ca^2+^ ion is bound, it always occupied Ca_2_ binding pocket. The third Ca^2+^ ion never occupied a stable binding pocket, as it is never coordinated by as much as six O-donors.

Throughout the metadynamics run, Ca_1_ kept its coordination shell as in the previous MD simulations (Table S1 and Figure S1,A). Due to the bias potential, Ca_2_ and Ca_4_ instead turned out to bind and unbind several times (Figure S1,B) exploring different configurations (Figure S2). Ca_2_ was either bound to D301, D302, D303, D312 and D323 (Figure 4) or it was solvated. Analogously, the third Ca^2+^ ion (Ca_4_ in Figure 4) was bound to D301, D302, D303, D304, E306, D312 and D323 in some of the stable conformations. Therefore, our metadynamics simulations confirmed that the residues identified by MD can play a role for Ca^2+^ binding. In addition, they further suggested a possible role of D301 and D303 for such binding.

### Computer-aided design of mutants with low-affinity for Ca^2+^

We attempted to identify mutations that could have an effect on Ca^2+^ binding. We constructed structural models in which putative key Asp and Glu residues among those identified in the above section were mutated to alanine. These are D301A, D302A, D303A, D304A, E306A, D312A and the double mutant E306A+D323A (see Table S2). Adducts with five Ca^2+^ ions underwent 20 ns of MD.

At the end of MD simulations, D301A bound only one Ca^2+^ ion (Table S2), while its 3-D structure remained similar to that of WT.

The Asp-rich domain bearing D304A mutation unfolded, possibly because D304 is the central residue of the domain and therefore responsible for its structural stability; all Ca^2+^ ions were partially or fully solvated (Table S2). Since the structural information of the channel is lacking, it is difficult to establish if the unfolding of D304A would occur in the real molecular surrounding, that is, when the Asp-rich domain is attached to the remaining bestrophin part.

Mutants D302A, D303A, E306A, and D312A resembled the WT as they bound at least two Ca^2+^ ions (Table S2). In fact, other residues contributed to Ca^2+^ coordination replacing the mutated ones.

Based on these results we concluded that D301A and D304A mutations have the largest effect on Ca^2+^ binding, while D302A, D303A, E306A, and D312A affect Ca^2+^ binding to a lesser extent.

In order to verify this, the experiments of the WT and mutant mBest2 were performed. The putative Ca^2+^-binding domain of mBest2 has almost the same sequence as that of hBest1 (Figure 1). To investigate whether the WT structures are affected by the differences, MD simulations were performed also on mBest2. As a result of MD simulations both models showed very similar behavior, with Ca_1_ and Ca_2_ that remained stably bound, while Ca_3_ and Ca_5_ were solvated. Ca_4_ bound more peptide O-donors in mBest2 than in hBest1. However, this difference should not be overestimated, since even starting from the same initial system (*e.g.* hBest1), running two independent MD simulations of the same length could result in slightly different events.

### Current recordings of wild-type and designed mutants

**Figure 5:**
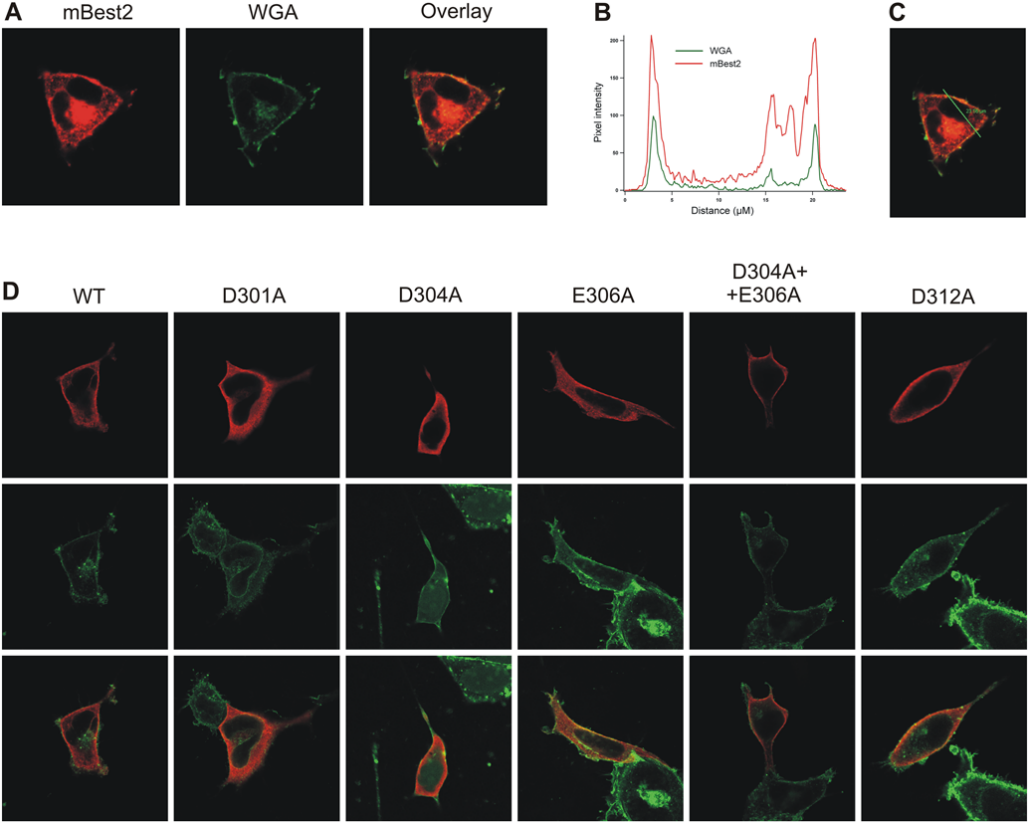
Localization of mBest2 and its mutants in HEK-293 cells. mBest2 was transfected into HEK-293 cells. 2–4 days after transfection, the cells were fixed, permeabilized and stained with antibody against mBest2, then visualized with Alexa-594-conjugated secondary antibody. Cells were also stained with Alexa-488 conjugated wheat germ agglutinin (WGA). Typical cell transfected with wild type mBest2 is shown in (A). Signal from mBest2 staining (*red*) is shown in the left, signal from WGA (*green*) from the same cell is in the middle. Overlay of both signals (right) indicates the expression of protein in plasma membrane and/or in close submembrane space. The distribution of mBest2 at the membrane was determined by comparing *red* and *green* fluorescence along a line drawn across a cell. Distribution of WGA- and mBest2- associated fluorescence along the line across the cell is shown in (B), line is shown in (C). Similar procedure was repeated for all five investigated mutations. Results for wild type (WT) mBest2, D301A, D304A, E306A, D304A+E306A and D312A mutations are compared in (D). Signal from staining against mBest2 (*red*) is shown in first row, signal from WGA (*green*) in the middle, overlay of both signals is in third row. Cells transfected with all investigated mutations showed similar distribution of signals, confirming increased localization of mutated proteins in plasma membrane and/or in close submembrane space. Non-transfected cells had negligible mBest2-associated fluorescence (data not shown).

The changes of Ca^2+^ coordination in the aforementioned mutations may affect the amplitude of bestrophin Ca^2+^-activated Cl^−^ currents, which can be experimentally measured. This was done here for mBest2. WT and mutants currents were compared after their heterologous expression in the HEK-293 cell line (Figure 5).

To determine if mutant proteins are delivered to the plasma membrane as WT, we used immunostaining with antibodies against mBest2 [11]. The WT protein showed increased localization in plasma membrane and/or in close sub-membrane space in a significant portion of transfected cells, although some fraction of protein was localized also intracellulary (Figure 5A). These results are similar to data previously published for hBest2 [8] and xBest2 [40]. The localization of the proteins was further investigated by co-staining with fluorescently labeled wheat germ agglutinin (WGA), a selective marker of glycosylated surface-expressed proteins. Co-localization of both signals, which indicates membrane expression of the protein, was observed. The distribution of mBest2-related signal and its co-localization with WGA in mutants were very similar to WT, indicating that the proteins were expressed and delivered to the plasma membrane (Figure 5). Consequently, changes of the measured current were caused by a modification of the channel functionality and not by the inability of the mutant proteins to reach the plasma membrane.

We then measured currents in HEK-293 cells transfected with mBest2 WT or mutants both in nominally 0 free Ca^2+^ level and in 22 µM free Ca^2+^ intracellular concentration. Currents were measured in the whole-cell voltage-clamp configuration applying voltage-steps of 20 mV from −100 mV to +100 mV from a holding potential of 0 mV. The current values at +60 mV were normalized to the capacitance of each cell and averaged.

**Figure 6:**
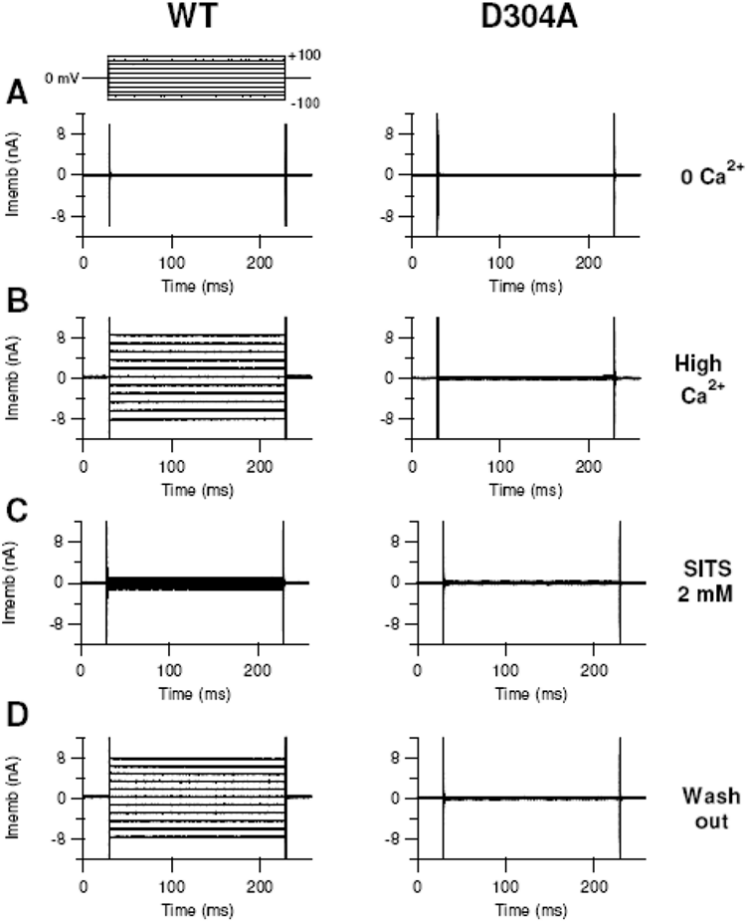
Currents in HEK-293 transfected cells. Currents in HEK-293 cells transfected with EGFP and WT mBest2 (left column) or D304A mutant (right column). Transfected cells were identified by the EGFP-expressed green fluorescence. Whole-cell voltage clamp recordings were obtained with pipette solutions containing nominally 0 (A) or 22 µM free Ca^2+^ (B–D). For each type of channel, recordings in B–D were obtained from the same cell, while traces in A are from different cells. Voltage steps of 200 ms duration were given 2 min after the reaching of the whole-cell configuration from a holding potential of 0 mV to voltages between −100 and +100 mV in 20 mV steps. In the left column, panels C and D show the reversible block of the WT current by 2 mM SITS, a Cl^−^ channel blocker. The mutant D304A did not show an appreciable current when Ca^2+^ was present in the intracellular solution (right column; see also Figure 7).

**Figure 7:**
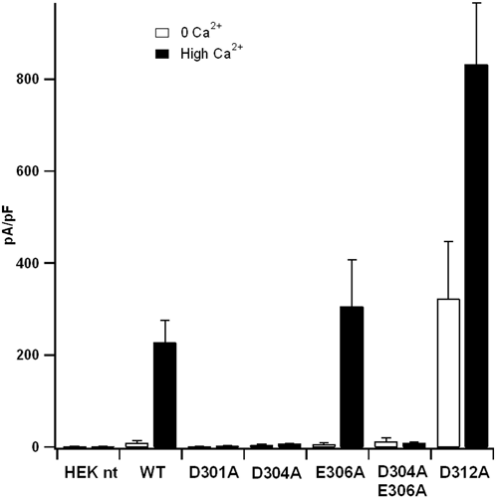
Comparison of current amplitudes from mutant and WT mBest2 Cl^−^ channels. Mean current densities from non-transfected cells (HEK nt) and cells transfected with EGFP and WT or mutated mBest2. The intracellular pipette solution contained nominally 0 (white bar) or 22 µM free Ca^2+^ (black bar). Data are shown as mean value±standard error of the mean. Each data represents the mean of at least five cells. Control experiments with non-transfected HEK-293 cells did not show any appreciable current. Mean current densities in the presence of 22 µM free Ca^2+^ of WT, E306A and D312A were significantly higher than those in nominally 0 Ca^2+^ (P<0.02; N = 5–13), whereas currents at the two Ca^2+^ levels were not statistically different for D301A, D304A and D304A+E306A (P>0.05, N = 5–20). Currents in the presence of 22 µM free Ca^2+^ of D301A, D304A, and D304A+E306A were substantially decreased compared with WT (P<0.002 for each group, N = 5–20). E306A had a mean value not significantly different from WT (P = 0.5; N = 7–8). D312A showed a current increase compared to WT both in the absence (P = 0.03; N = 9–13) and in the presence of Ca^2+^ (P = 0.003; N = 6–8).

The WT current in nominally 0 Ca^2+^ was significantly smaller than that in 22 µM Ca^2+^ concentration (Figures 6–7). This shows that Ca^2+^ ions activated a current in agreement with previous results [5], [11]. Most of the Ca^2+^-induced current was reversibly blocked by the Cl^−^ channel blocker SITS indicating the current is indeed carried by Cl^−^.

In contrast, D304A and D301A produced negligibly small currents in both Ca^2+^ levels (Figure 7). Two other mutants, E306A and D312A, produced currents: whilst the amplitude of the current of E306A was similar to that of WT (Figure 7), D312A showed a somehow unexpected result. It produced a substantial amount of current in the nominally 0 Ca^2+^ concentration (Figure 7). In 22 µM Ca^2+^ concentration, the current significantly increased and was larger than that of WT. The current was also blocked by SITS in both Ca^2+^ levels, indicating that it is a Cl^−^ current (data not shown). Therefore, the D312A mutation not only allowed a Ca^2+^-activated Cl^−^ current, but it also modified channel gating increasing its current also in the absence of Ca^2+^.

Finally, the double mutant D304A+E306A abolished the current, similarly to the single mutant D304A (Figure 7).

Experiments showed that WT, E306A and D312A mutants allowed a substantial Ca^2+^-activated current, whereas D301A and D304A mutants produced a negligibly small Ca^2+^-activated current. These results were fully consistent with our theoretical predictions.

## Discussion

Understanding the interactions between the Ca^2+^ ions and the Asp-rich domain in bestrophins is an important step in the investigation of the physiological role of these proteins, because they are putatively involved in the Ca^2+^-activated Cl^−^ current in some epithelial cells [12]–[14]. In addition, it seems to be also important for understanding the pathophysiology of the Best disease, since the Asp-rich domain is a “hot spot” for mutations causing the illness [19], [20]. At present nothing is known about the underlying structural features of the bestrophins.

We propose that the Asp-rich domain located at the C-terminal region of bestrophins can contribute to Ca^2+^ binding [10], due to its similarity with other Ca^2+^-binding domains, as the Ca^2+^ bowl in BK_Ca_ [26], [30], [32] and T3 repeats of thrombospondins [31], [36], [37]. This statement is consistent with previous experimental data, which show that mutations of putative Ca^2+^ ligands in bestrophin Asp-rich domain affect dramatically the current [4], [22].

In order to identify negatively charged residues in the domain involved in Ca^2+^ binding, we adopted a multifaceted strategy involving homology modelling, MD simulations, immunostaining and electrophysiological experiments of WT and mutated bestrophins.

Theoretical data suggest that not all Asp and Glu residues have equal importance in Ca^2+^ binding. Simulations of D301A and D304A mutants point to the key role of these residues for binding. Measures of Ca^2+^-sensitive Cl^−^ current of WT and mutant bestrophins expressed in HEK-293 cells show that D301A and D304A mutations do actually affect the functionality of the channel, dramatically reducing the Ca^2+^-activated current amplitude. These findings are fully consistent with our theoretical predictions.

Other residues emerge to have relevance for Ca^2+^ binding, albeit smaller, (Table S1). These include E306 and D312. In our experiments, the mutation of these residues to alanine does not cause reduction in the current in agreement with the simulations, where other O-donors can replace the aforesaid residues in the Ca^2+^ coordination shell.

In conclusion, simulations suggest that at least two Ca^2+^-binding sites could be present in the Asp-rich domain of bestrophins. Furthermore, electrophysiological experiments show that mutations predicted by our modeling have an impact on the function, decreasing the Ca^2+^-activated current amplitude and confirming the importance of the bestrophin Asp-rich domain in Ca^2+^-dependent activation of the channel.

Finally, the presence of Asp repeats also in thrombospondins and BK_Ca_ channels suggests that they could represent a general motif for Ca^2+^ binding in different classes of proteins.

## Materials and Methods

### Bioinformatics

The sequences of the human TSP-1 T3_5-7_ repeats (PDB code: 1UX6) [36] were aligned with the Ca^2+^ bowl from human and mouse BK_Ca_ channels [26]–[30], [41], [42] and the Asp-rich domains of hBest1 [1], [2], mBest1 [2], hBest2 [43], mBest2 [44], hBest3 [43], mBest3 [45], hBest4 [43], ceBest1 [4], dvBest1 [46], dmBest2 [47] and dmBest4 [47] (Swiss-Prot entries and notations are reported in the legend of Figure 1). The alignment was initially performed with ClustalW [48] and then slightly refined to avoid apolar residues in Ca^2+^ binding positions. Sequence similarity was determined considering amino acid pairs having positive values in BLOSUM62 [49] substitution matrices. The homology models of hBest1 and mBest2 Asp-rich domains from W287 to D323 were built with the Modeller 6v2 program [50], [51], using the crystal structure of the TSP-1 T3_6_ motif as a template (PDB code: 1UX6) [36]. In the case of hBest1, models were generated where five to three Ca^2+^ ions present in the template were kept. These are: (i) M5, with five Ca^2+^ ions coordinated. (ii) M4 is composed by four Ca^2+^ ions which are Ca_1_, Ca_2_, Ca_4_ and Ca_5_. (iii) M3′ and M3″ are including three Ca^2+^ ions, Ca_1_, Ca_2_, Ca_4_ or Ca_1_, Ca_2_, Ca_5_, respectively.

### MD simulations

Models of the Asp-rich domain of hBest1 were inserted in a box of edges 49, 50 and 53 Å filled with ∼4,100 water molecules [52]. Cl^−^ (three in M5, one in M4) or Na^+^ (one in M3′ and M3″) counterions were added to ensure systems charge neutrality. The force fields for the protein, ions and water molecules were the Amber parm99 [53], [54] and TIP3P [52] force fields, respectively.

For M5, energy minimization of water and counterions was performed with position restraints on the Asp-rich domain and Ca^2+^ ions, with a force constant of value 25.0 kcal/mol Å^−2^. After the first 300 cycles of steepest descent minimization, conjugate gradient method was used until 50,000 cycles. Then steepest descent minimization of the system without restraints was performed for another 150 cycles, following by conjugate gradient minimization until 50,000 cycles. After that, constrained solute and Ca^2+^ ions underwent 60 ps of linear heating MD from 0 K till 300 K. Restraints on Asp-rich domain and Ca^2+^ ions corresponded to a force constant of 5.0 kcal/mol Å^−2^. A time step of 2 fs was used. Finally, the system underwent NPT MD simulations for 75 ps at 1 fs time step. The system was maintained at the reference pressure and temperature of 1 bar and 300 K, respectively, by coupling the system to a Berendsen thermostat and barostat [55]. A cut-off of 9 Å was used for calculating non-bonded interactions.

Finally, unrestrained MD simulations were carried out for an overall length of 70 ns. A time step of 2 fs was used. All bond lengths were kept fixed applying the LINCS algorithm [56]. Temperature (300 K) and pressure (1 bar) were kept constant as aforementioned. Periodic boundary conditions were applied treating long-range electrostatic interactions with the particle-mesh Ewald technique using a short-range cutoff of 10 Å [57]. The same cutoff was used for the van der Waals interactions. Pair lists were updated every 20 steps.

Models with four or three Ca^2+^ ions were built starting from the MD snapshot of M5 at 9 ns (after the initial relaxation) by manually removing one or two ions (see above). In M4, M3′ and M3″ models Ca_3_ was always removed since it is immediately solvated during the simulation of M5 (Figure 3). M4, M3′ and M3″ underwent then MD simulations for 20 ns (M4) and 35 ns (M3′, M3″), respectively, using exactly the same protocol as that used for the M5 model.

The models of the Asp-rich domain mutants (D301A, D302A, D303A, D304A, E306A, D312A, E306A+D323A) were built, based on the M5 model, using the program Molden [58]. They underwent the same protocol of minimization and relaxation of the WT followed by 20-ns MD runs.

The model of mBest2 Asp-rich domain underwent the same protocol of minimization and relaxation of hBest1, followed by a 60-ns MD run.

All the systems underwent minimization using Amber 8.0 program [59] and then MD simulations using the Gromacs 3.2.1 program [60], [61]. Trajectory analysis was performed by using the VMD 1.8.6 program [62].

### Metadynamics

A 60-ns long metadynamics simulation was performed by biasing two collective variables (CVs) with a history-dependent potential. These are the coordination numbers of Ca_2_ and Ca_4_ to Asp and Glu residues of the Asp-rich domain. They were defined as the number of contacts shorter than 4.3 Å among one Ca^2+^ ion and all carboxylate carbons of the studied domain:
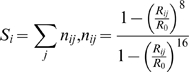
where *i* runs over Ca_2_ and Ca_4_, *j* runs over all carboxylate carbons, *R_ij_* is the distance between the two atoms, *R*_0_ = 4.3 Å is a cutoff distance chosen in order to discriminate between bonded and non-bonded Ca^2+^ ions (this cutoff distance ensures that Ca^2+^ ion is considered bonded when it is at most 3 Å away from carboxylate oxygen atoms (see *e.g.* [38]), and *n_ij_* is a function switching smoothly between 1 (bonded Ca^2+^ ion) and 0 (non-bonded Ca^2+^ ion). Similar CVs have been employed in a previous study of Ca^2+^-binding polymers [63]. The 2-D Gaussians forming the metadynamics bias potential have width σ_1_ = σ_2_ = 0.2 (along S_1_ and S_2_ respectively) and a height of 0.2 kcal/mol. A new Gaussian is added along the trajectory every 1 ps. Metadynamics is implemented in a modified version of the Gromacs-3.3.1 package [39]. The code has been kindly provided by Alessandro Laio.

### Site-specific mutations of mBest2 and heterologous expression

The clone of mBest2 in pCMV-Sport6 mammalian expression plasmid was obtained from the RZPD (Berlin, Germany) collection (Deutsches Ressourcenzentrum für Genomforschung). Site-specific mutations of mBest2 were made using a PCR-based site-directed mutagenesis kit (Quiagen). Mutations were confirmed by DNA sequencing.

mBest2 and its mutants were transfected into HEK-293 cells along with a vector that expressed EGFP (pEGFP; Invitrogen) at a 1∶8 ratio using Fugene-6 transfection reagent (Roche). 2 days after transfection, cells were dissociated and replated. Transfected cells were identified by EGFP fluorescence and used for patch clamp experiments within 3 days after transfection.

### Immunofluorescence and confocal microscopy

To test successful expression of proteins, as well as their spatial distribution in HEK-293 cells, we used co-staining of antibodies against mBest2 and labeled wheat germ agglutinin (WGA) as a marker of glycosylated surface-expressed proteins – a method previously used to show localization of proteins in cell membrane and/or close submembrane space [40], [64].

Transfected cells (without the EGFP expressing vector) were fixed with 4% paraphormaldehyde for 20 min, washed, incubated with 5 µg/ml WGA conjugated to Alexa Fluor 488 (Invitrogen) for 20–30 min, incubated for 30 min in blocking solution containing 1% BSA (Sigma), 0.1% Triton X-100 (Sigma) and 1% FCS (Sigma), then for 2 hours with an antibody against mBest2 [11] (diluted in 1∶500 ratio with blocking solution), washed extensively and incubated with a A594-conjugated anti-rabbit secondary antibody (Invitrogen, diluted 1∶500 in blocking solution) for 1 hour, washed and mounted with Vectashield (Vector Laboratories, Burlingame, CA). Immunoreactivity was analyzed by confocal microscopy with a Leica TCS SP2 confocal microscope. All incubations were made at room temperature; all substances were diluted in PBS, unless otherwise indicated.

### Electrophysiological recordings

Currents were measured with an Axopatch 200B patch-clamp amplifier (Molecular Devices, CA, USA) in the whole-cell voltage-clamp mode. The amplifier was controlled via a Digidata 1440A (Molecular Devices, CA, USA).

Patch pipettes were made using borosilicate capillaries (WPI, Sarasota, Florida, USA) and pulled with a Narishige PP83 puller (Narishige, Tokyo, Japan), using a double stage pull. The pipette resistance was 2–4 MΩ. Data were sampled at 20 kHz and low-pass filtered at 10 kHz. Acquisition and storage of data were performed with the PClamp 10 software (Axon Instruments, CA, USA). Data analysis and figures were made with the Clampfit 10 software (Axon Instruments, CA, USA) and Igor 6.0 software (Wavemetrics, Lake Oswego, OR, USA).

The holding potential was 0 mV and a voltage protocol from −100 mV to +100 mV, with 20 mV steps of 200 ms duration was applied (starting 2 minutes after reaching the whole-cell configuration). Cell capacitance was estimated from a 10 mV step test protocol in the presence of the extracellular Cl^−^ current blocker SITS. Current densities were calculated normalizing the current recorded at +60 mV to the capacitance of each cell. Data are shown as mean±standard error of the mean. N is the number of cells. Statistical significance was determined using un-paired *t*-tests. *P* values<0.05 were considered significant.

The standard extracellular solution contained (in mM): 140 NaCl, 5 KCl, 2 CaCl_2_, 1 MgCl_2_, and 10 HEPES, 10 Glucose, pH = 7.4. The pipette solution contained (in mM): 130 CsCl, 2 MgCl_2_, 10 HEPES, 5 EGTA without added Ca^2+^ for the nominally 0 Ca^2+^ solution, or with 5 mM CaCl_2_ for the 22 µM Ca^2+^ free pipette solutions (free Ca^2+^ was calculated with the program WinMAXC, C. Patton, Stanford University, Palo Alto, CA, USA). pH was adjusted to 7.3 by adding HCl or CsOH. The osmolarity was adjusted with sucrose to 310 or 300 mOsm for the extracellular and intracellular solution, respectively.

To block Cl^−^ currents, the extracellular blocker 4-acetamido-4′-isothiocyanato-stilben-2,2′-disulfonate (SITS) was directly dissolved in the extracellular solution at 2 mM. All chemicals were purchased from Sigma. All experiments were carried out at room temperature (20–22°C).

## Supporting Information

Text S1 Test of the accuracy of the force field used in the MD simulations
(0.11 MB PDF)

Table S1 Percentage of time during which specific residue binds Ca^2+^ ions. Percentages were averaged over all MD simulations of all the studied models. Ca_3_ is not reported as it is never bound to the protein. “bb” indicates that the backbone carbonyl oxygen is involved in the binding.
(0.04 MB DOC)

Table S2 MD simulations of the M3′ model and its alanine mutants. Ca^2+^ protein coordination numbers in WT and the investigated mutants of the Asp-rich domain, as observed after 20 ns of MD simulations. First number refers to the number of peptide O-donors, whereas the second refers to the number of coordinated waters. Ca^2+^ ions were considered bound if they were coordinated with at least 5 protein O-donors and not more than 2 water molecules. Bound ions are highlighted (see text).
(0.04 MB DOC)

Figure S1 Metadynamics simulation of M3′ model. A. Number of peptide O-donors (red) and water molecules (blue) coordinating Ca_1_ through the metadynamics simulations of the Asp-rich domain of hBest1. As in standard MD, Ca_1_ is stably bound to the protein for most of the time. B. Number of peptide O-donors coordinating Ca_2_ (black) and Ca_4_ (green) plotted as a function of the metadynamics simulation time.
(1.19 MB TIF)

Figure S2 Most populated conformations of hBest1 Asp-rich domain as sampled by the metadynamics simulations. Asp/Glu residues coordinating Ca^2+^ ions are depicted. Ca^2+^ ions are represented as spheres (green: Ca_1_, orange: Ca_2_, yellow: Ca_4_) and numbered according to their binding positions as represented in the Figure 2 and S1.
(2.44 MB TIF)
