# Supplementary material for: Regulation of Bestrophins by Ca2+: A Theoretical and Experimental Study
Source: PLoS One. 2009 Mar 5;4(3):e4672. doi: 10.1371/journal.pone.0004672 (PMC2650406; doi:10.1371/journal.pone.0004672)
Supplement: Text S1 — Test of the accuracy of the force field used in the MD simulations (0.11 MB PDF) [file pone.0004672.s001.pdf]

# Regulation of Bestrophins by $Ca^{2+}$: a Theoretical and Experimental Study

**Agata Kranjc[1], Federico W. Grillo[1], Juraj Rievaj[1], Anna Boccaccio[1,2], Fabio Pietrucci[1], Anna Menini[1,2], Paolo Carloni[1,2,3], Claudio Anselmi[1,3]***

**1** International School for Advanced Studies (SISSA/ISAS), Via Beirut 2-4, I-34014, Trieste, Italy, **2** Italian Institute of Technology (IIT), SISSA-Unit, Via Beirut 2-4, I-34014, Trieste, Italy, **3** CNR-INFM-DEMOCRITOS Modeling Center for Research in Atomistic Simulation, Via Beirut 2-4, I-34014, Trieste, Italy.

## Text S1

### Test of the accuracy of the force field used in the MD simulation.

Structures and energetic of $Ca^{2+}$-binding proteins are difficult to reproduce with current classical force fields [1,2]. Factors, which limit the accuracy, include the lack of polarizability and charge transfer effects [3]. Here we tested the accuracy of three commonly used force fields: the Aqvist [4], the Bradbrook [5] and the AMBER [6,7] force fields. We performed 20-ns MD calculations of TSP-1 $T3_6$ for which the X-ray structure is available [8] and compared X-ray and MD structures.
TSP-1 $T3_6$ is a 39 amino acids long loop resembling EF hands motif in the spacing of acidic side chains [9,10]. It binds five $Ca^{2+}$ ions by eleven Asp and two Asn residues (See Figure). Here we considered a model in which all five ($Ca_{1-5}$) calcium ions were present. The TSP-1 $T3_6$ domain was inserted in a box of edges 46, 51 and 49 Å filled with ~3700 water molecules and 2 $Cl^-$ counterions. Computational details for the relaxation and unrestrained MD simulations were the same as reported in the main text of the paper.
In the three simulations, the final structure turned out to be rather similar to the X-ray structure: the RMSD values calculated onto the N-terminal and C-terminal halves are indeed equal to 3.2 Å and 2.4 Å, respectively (see table below) and $Ca_{1-4}$ remained bound. However, the total backbone RMSD from the X-ray structure was relatively large because of conformational changes of $Ca_5$–binding groups (D880 and D887) upon $Ca_5$ solvation after few ns (~2 ns for Aqvist- and Bradbrook-based MD, ~1.3 ns for the AMBER-based MD). The loss of the $Ca^{2+}$ ion might be caused by several factors, including the limitations of the accuracy of the force field describing the $Ca^{2+}$ ions [2] and/or the absence of electrostatic packing forces in the simulation with respect to the crystal, along with a difference in temperature (the simulation was run at 300 K, the crystal structure was solved at 100 K). Force-matching methods, which were already applied by us for other metal-containing systems [11], might be used to further address this issue, which is however beyond the

scope of the present paper and the severe computational resources that would be needed prevent the application of these methods to the present problem. In all of the other calculations presented in this work we have indeed used the AMBER force field.

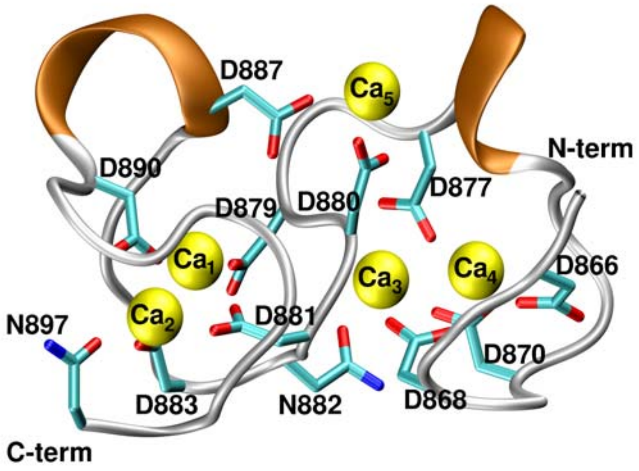


**Figure. Crystal structure of TSP-1 T3$_6$ domain.** Five $Ca^{2+}$ ions are bonded to the Asp/Asn residues. For clarity, $Ca^{2+}$ ions are indicated as $Ca_{1\text{-}5}$ according to their binding positions. The same labels are used throughout the text in the model of the hBest1 Asp-rich domain.

Because of its limitations, we used molecular modeling to identify possible $Ca^{2+}$-binding conformations of the Asp-rich domain and alanine mutations, which could affect $Ca^{2+}$ binding. No attempts were made to predict the structure of disease-linked mutants [12] and/or mutants for which electrophysiological experiments have been previously carried out [13] .

N-term corresponds to residues from A865 to H878, whereas C-term corresponds to residues from D879 to N897.

**Table.** TSP-1 T3$_6$ backbone RMSD values relative to the X-ray structure after 20-ns MD simulations based on three different force fields.

| | Aqvist [4] | Bradbrook [5] | AMBER [6,7] |
|---|---|---|---|
| Total RMSD (Å) | 3.5 | 5.2 | 5.5 |
| RMSD of N-term (Å) | 3.5 | 3.8 | 3.2 |
| RMSD of C-term (Å) | 2.6 | 4.3 | 2.4 |

## Reference List


1. Costa V, Carloni P (2003) Calcium Binding to the Transmembrane Domain of the Sarcoplasmic Reticulum Ca2+-ATPase: Insights from Molecular Modeling. Proteins 50: 104-113.

2. Marchand S, Roux B (1998) Molecular dynamics study of calbindin $D_{9k}$ in the apo and singly and doubly calcium-loaded states. Proteins 33: 265-284.

3. Dal Peraro M, Raugei S, Carloni P, Klein ML (2005) Solute-solvent charge transfer in aqueous solution. Chemphyschem 6: 1715-1718.

4. Aqvist J (1990) Ion water interaction potentials derived from free-energy perturbation simulations. J Phys Chem 94: 8021-8024.

5. Bradbrook GM, Gleichmann T, Harrop SJ, Habash J, Raftery J et al. (1998) X-ray and molecular dynamics studies of concanavalin-A glucoside and mannoside complexes - relating structure to thermodynamics of binding. J Chem Soc -Faraday Trans 94: 1603-1611.

6. Ponder JW, Case DA (2003) Force fields for protein simulations. Adv Protein Chem 66: 27-85.

7. Wang J, Cieplak P, Kollman PA (2000) How well does a restrained electrostatic potential (RESP) model perform in calculating conformational energies of organic and biological molecules? J Comput Chem 21: 1049-1074.

8. Kvansakul M, Adams JC, Hohenester E (2004) Structure of a thrombospondin C-terminal fragment reveals a novel calcium core in the type 3 repeats. EMBO J 23: 1223-1233.

9. Misenheimer TM, Mosher DF (1995) Calcium ion binding to thrombospondin 1. J Biol Chem 270: 1729-1733.

10. Chen H, Deere M, Hecht JT, Lawler J (2000) Cartilage oligomeric matrix protein is a calcium-binding protein, and a mutation in its type 3 repeats causes conformational changes. J Biol Chem 275: 26538-26544.

11. Spiegel K, Magistrato A, Maurer P, Ruggerone P, Rothlisberger U et al. (2008) Parameterization of azole-bridged dinuclear platinum anticancer drugs via a QM/MM force matching procedure. J Comput Chem 29: 38-49.

12. Horling F (2006) The VMD2 Database (http://www-huge.uni-regensburg.de/VMD2_database/index.php?select_db=VMD2).

13. Hartzell HC, Qu Z, Yu K, Xiao Q, Chien LT (2008) Molecular physiology of bestrophins: multifunctional membrane proteins linked to best disease and other retinopathies. Physiol Rev 88: 639-672.
