# Supplementary material for: Regulation of Bestrophins by Ca2+: A Theoretical and Experimental Study
Source: PLoS One. 2009 Mar 5;4(3):e4672. doi: 10.1371/journal.pone.0004672 (PMC2650406; doi:10.1371/journal.pone.0004672)
Supplement: Table S1 — Percentage of time during which specific residue binds Ca2+ ions. Percentages were averaged over all MD simulations of all the studied models. Ca3 is not reported as it is never bound to the protein. “bb” indicates that the backbone carbonyl oxygen is involved in the binding. (0.04 MB DOC) [file pone.0004672.s002.doc]

**Table S1** Percentage of time during which specific residue binds Ca^2+^ ions.

|  | Ca_1_ | Ca_2_ | Ca_4_ | Ca_5_ |
| --- | --- | --- | --- | --- |
| **Glu292** |  |  | 56% |  |
| **Asn296** |  |  | 41% |  |
| **Glu300** |  |  | 100% |  |
| **Asp302** | 100% |  |  |  |
| **Asp303** |  | 31% | 63% | 88% |
| **Asp304 Asp304 (bb)** | 100% 71% | 100% ---- |  |  |
| **Glu306** | 100% | 93% |  |  |
| **Asp312 Asp312 (bb)** | 100% ---- | 36% ---- |  | 87% 71% |
| **Gln316** |  | 25% |  | 50% |
| **Ser318 Ser318 (bb)** |  | 44% 73% |  |  |
| **Ala321 (bb)** |  | 85% |  |  |
| **Asp323** |  | 100% |  |  |

Percentages were averaged over all MD simulations of all the studied models. Ca_3_ is not reported as it is never bound to the protein. “bb” indicates that the backbone carbonyl oxygen is involved in the binding.
