# Supplementary material for: Regulation of Bestrophins by Ca2+: A Theoretical and Experimental Study
Source: PLoS One. 2009 Mar 5;4(3):e4672. doi: 10.1371/journal.pone.0004672 (PMC2650406; doi:10.1371/journal.pone.0004672)
Supplement: Table S2 — MD simulations of the M3′ model and its alanine mutants. Ca2+ protein coordination numbers in WT and the investigated mutants of the Asp-rich domain, as observed after 20 ns of MD simulations. First number refers to the number of peptide O-donors, whereas the second refers to the number of coordinated waters. Ca2+ ions were considered bound if they were coordinated with at least 5 protein O-donors and not more than 2 water molecules. Bound ions are highlighted (see text). (0.04 MB DOC) [file pone.0004672.s003.doc]

**Table S2** MD simulations of the M3’ model and its alanine mutants.

Ca^2+^ protein coordination numbers in WT and the investigated mutants of the Asp-rich domain, as observed after 20 ns of MD simulations. First number refers to the number of peptide O-donors, whereas the second refers to the number of coordinated waters. Ca^2+^ ions were considered bound if they were coordinated with at least 5 protein O-donors and not more than 2 water molecules. Bound ions are highlighted (see text).

|  | **Ca_1_** | **Ca_2_** | **Ca_3_** | **Ca_4_** | **Ca_5_** | **EXP** |
| --- | --- | --- | --- | --- | --- | --- |
| **WT (0 ns)** | **6+0** | **6+1** | 4+4 | **6+1** | 5+2 | ---- |
| **WT (20 ns)** | **6+1** | **7+1** | 5+3 | 4+3 | 5+3 | ---- |
| **D301A** | 5+3 | **5+2** | 5+3 | 5+3 | 3+4 | **no current** |
| **D302A** | **6+2** | **6+2** | 2+4 | 5+3 | 2+6 | ---- |
| **D303A** | **7+1** | **7+1** | 3+5 | 5+3 | 2+6 | ---- |
| **D304A** | 4+4 | 5+3 | 0+8 | 3+5 | 2+6 | **no current** |
| **E306A** | **6+1** | **8+0** | 4+4 | **6+1** | 4+4 | **current** |
| **D312A** | **6+2** | **7+1** | 2+4 | 4+4 | 0+7 | **current** |
