# Supplementary figures and images for: Regulation of Bestrophins by Ca2+: A Theoretical and Experimental Study
Source: PLoS One. 2009 Mar 5;4(3):e4672. doi: 10.1371/journal.pone.0004672 (PMC2650406; doi:10.1371/journal.pone.0004672)

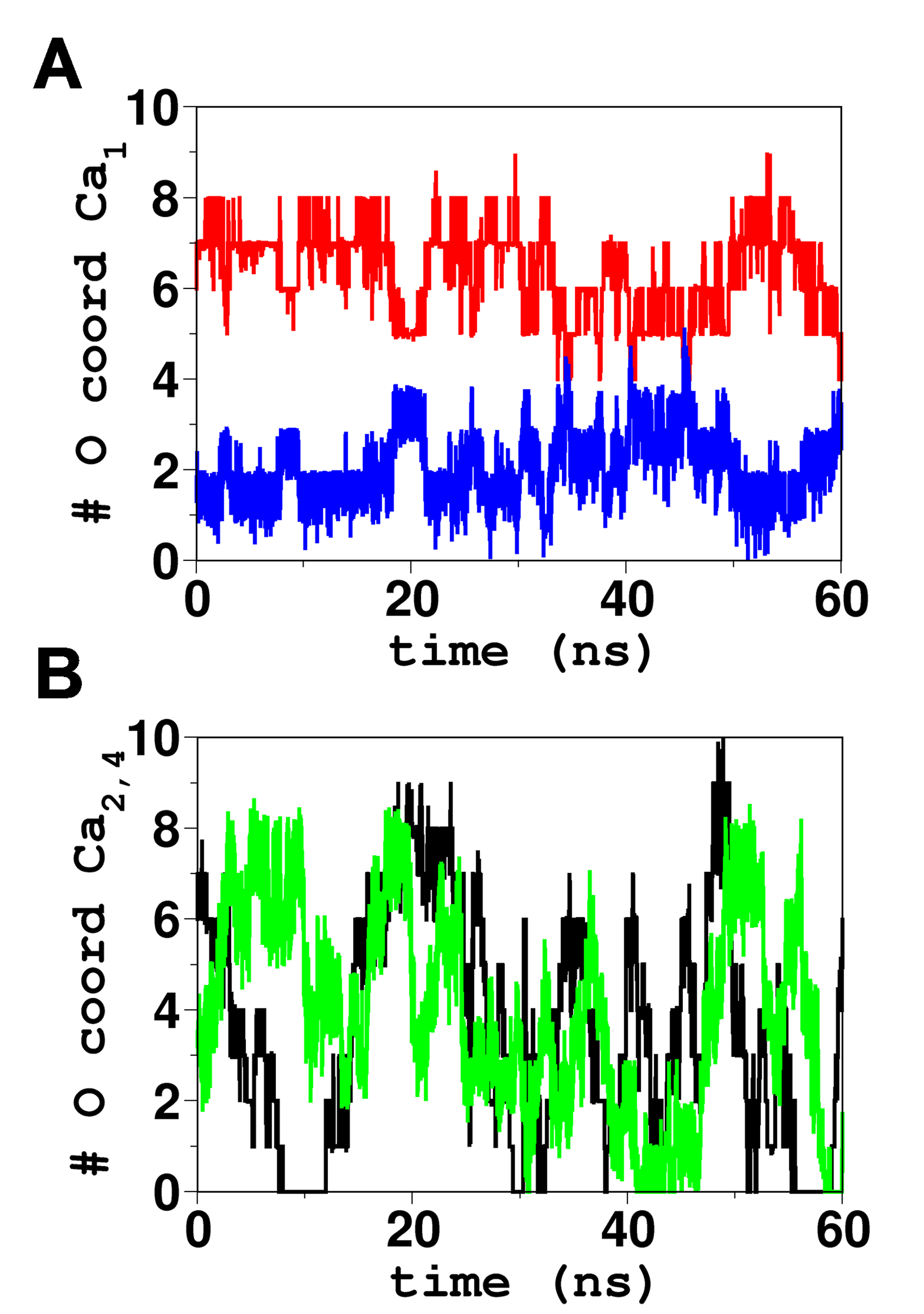

Supplement: Figure S1 — Metadynamics simulation of M3′ model. A. Number of peptide O-donors (red) and water molecules (blue) coordinating Ca1 through the metadynamics simulations of the Asp-rich domain of hBest1. As in standard MD, Ca1 is stably bound to the protein for most of the time. B. Number of peptide O-donors coordinating Ca2 (black) and Ca4 (green) plotted as a function of the metadynamics simulation time. (1.19 MB TIF) [file pone.0004672.s004.tif]

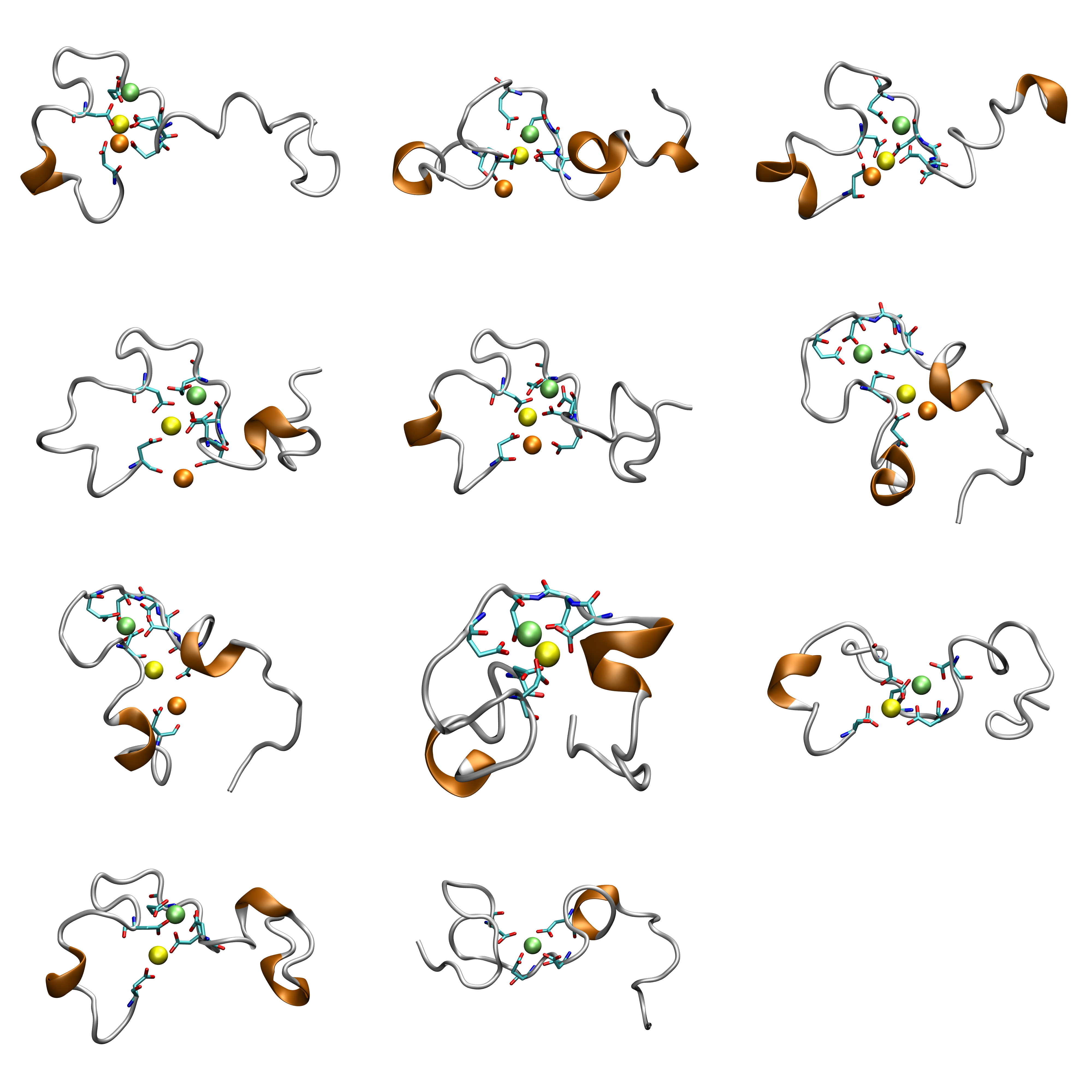

Supplement: Figure S2 — Most populated conformations of hBest1 Asp-rich domain as sampled by the metadynamics simulations. Asp/Glu residues coordinating Ca2+ ions are depicted. Ca2+ ions are represented as spheres (green: Ca1, orange: Ca2, yellow: Ca4) and numbered according to their binding positions as represented in the Figure 2 and S1. (2.44 MB TIF) [file pone.0004672.s005.tif]
